# Radiation recall dermatitis following letrozole administration in patient with a remote history of radiation therapy

**DOI:** 10.1038/s41523-021-00271-3

**Published:** 2021-05-26

**Authors:** Evan Sweren, Pathik Aravind, Robert Dembinski, Catherine Klein, Mehran Habibi, Michelle L. Kerns

**Affiliations:** 1 Department of Dermatology, Johns Hopkins University School of Medicine, Baltimore, MD USA; 2 Department of Surgery, Johns Hopkins Hospital, Bayview Campus, Baltimore, MD USA

**Keywords:** Cancer microenvironment, Breast cancer

## Abstract

We report the case of letrozole-induced radiation recall dermatitis (RRD) in a patient with a remote history of radiation therapy. There is only one previously known case of RRD triggered by letrozole in a patient with a recent (<3 month) history of radiation. Previously, only four other cases of aromatase-inhibitor-induced RRD have been reported. This case is significant for cancer care teams considering personalized treatments. In addition, improved long-term outcomes in cancer patients may lead to increases in and underdiagnoses of RRD. Likewise, RRD is patient specific, exacerbating health concerns, and can be difficult to recognize without proper awareness, documentation, and classification of triggering drugs. The authors hope to address these issues in this report.

## Introduction

Radiation recall dermatitis (RRD) is a localized drug-induced inflammatory skin reaction occurring in a previously irradiated site months to years after discontinuation of ionizing radiation exposure. D’Angio et al. first described RRD in 1959 in association with actinomycin-D^1^. Since then, numerous pharmacological agents have been implicated as potential RRD triggers, with each trigger drug and risk factors being patient specific and unpredictable^2,3^. Clinical signs of RRD include erythema, pruritus, pain, desquamation, edema, vesiculation, necrosis, ulceration, and hemorrhage and can arise hours to months after initiation and even discontinuation of triggering medicines^4^. Recognition of RRD is of particular relevance for cancer care teams to avoid misdiagnoses and inappropriate treatments given anticancer medications comprise around 20–30% of RRD cases^5^. We report the case of letrozole (a selective aromatase inhibitor)-induced RRD in a 78-year-old woman nine years after ionizing radiation exposure, the longest known radiation-RRD gap for letrozole, the second reported case for this drug, and the first exclusively independent of potential ARD confounders^6^.

## Results

### Case history and presentation

A 78-year-old woman with cancer at age 58, 69, and 78 presented to the emergency room with fevers, chills, malaise, and painful erythema of the left chest wall. A review of her history indicated that the cancer at age 58 was left-sided ductal carcinoma in situ treated with lumpectomy, followed by 5 years of adjuvant tamoxifen without radiation. The cancer at age 69 was left-sided lobular carcinoma in situ with microinvasion, which was treated with excision of the targeted area and adjuvant intensity modulated radiation therapy (IMRT) at a dose of 42.56 Gy to the whole breast (mixed energy) and an additional 8.1 Gy electron boost to the surgical bed with (12-MeV energy); total cumulative dose was 50.66 Gy. The pathology was microinvasive lobular carcinoma, arising in a background of multifocal lobular carcinoma in situ (LCIS), ER- (0%), PR - (0%), HER-2 negative (1+), Ki-67 10–15%. The LCIS was patchy ER + (5–10%), patchy PR + (5–10%).

Most recently, her cancer at age 78 was a 2.5 cm invasive lobular carcinoma in her left breast that was treated with a simple mastectomy and sentinel lymph node biopsy. The cancer was T2N0Mx, grade 2, ER + (>95%, strong), PR focally + (1–5%), HER-2/neu equivocal by IHC (2+), not amplified by FISH, HER-2: D 17Z1 ratio 0.9, average HER-2 signals per nucleus 2.6. Ki-67 was 50–60%. Four left axillary lymph nodes were negative. Though the patient developed a rash around the incision accompanied with fever and chills following surgery, the rash completely resolved with Augmentin by the second week post operation despite being unresponsive to Bactrim. Letrozole was then started at a dose of 2.5 mg orally.

In the emergency room, ~2 weeks following the initiation of letrozole, the patient reported a two-day history of fevers, chills, malaise and painful, warm erythema localized to a 20 cm × 10 cm area of the left chest wall. On exam, her surgical incision appeared to be well healed. No fluctuance or induration was appreciated. Laboratory values were remarkable for leukocytosis (WBC 13.41). No imaging was performed. She was diagnosed with cellulitis and treated with a seven-day course of the antibiotic Bactrim. At follow up evaluation, the patient’s fever was found to have resolved, and her WBC had normalized. However, given persistence of cutaneous symptoms, the patient was started on a course of Augmentin as well as treatment with topical Clotrimazole for a possible fungal infection. Her rash continued to darken and became more violaceous in color. After ~2 weeks with no improvement of the skin eruption, despite these interventions, the patient was referred to dermatology for further evaluation.

**Fig. 1:**
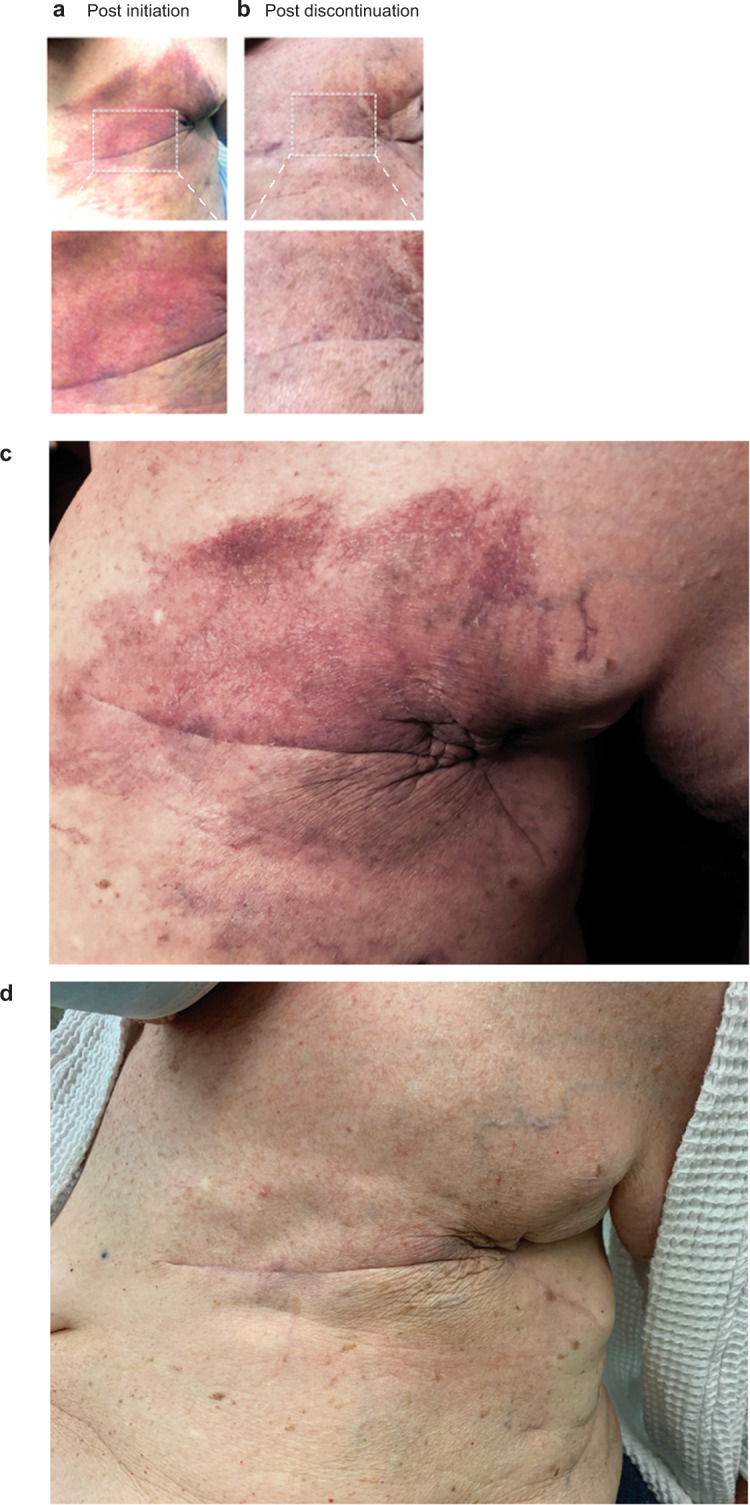
Letrozole induces radiation recall dermatitis (RRD). **a**, **c** Irregular erythematous purpuric patch with telangiectases involving the left chest wall, extending from the sternum to the left axilla at site of previous radiation 3 weeks after initiation of letrozole. **b**, **d** Resolution of skin eruption 3 months after discontinuation of letrozole.

Physical examination at her initial presentation to dermatology clinic revealed an irregular, erythematous to violaceous patch with telangiectases involving the left chest wall and extending from the sternum to the left axilla (Fig. 1a). No induration or tenderness was appreciated. There was a striking localization of the discoloration to previously irradiated area nearly 10 years prior. Our patient denied any other trauma to the site other than her recent surgery. Given the clinical history and physical findings, the patient was diagnosed with RRD. Follow up was recommended in 4–6 weeks for a biopsy unless the rash had improved. With the initiation of letrozole, the patient had also developed malaise, nausea, hair thinning, fatigue, and mood disturbances. In light of the occurrence of RRD as well as adverse side effects, the decision was made by the oncology team to cease letrozole treatment. Within 3 days, the patient reported improved feelings of non-cutaneous disturbances. Over 3 months of discontinued letrozole, her RRD has resolved, and the discoloration of the left chest wall has faded with almost no skin involvement (Fig. 1b). Currently, alternative systemic treatments are being considered for this patient.

## Discussion

RRD is a rare phenomenon with a significant impact on cancer patients, whose exact frequency is unknown due to misdiagnoses and underreporting; it has been suggested to be anywhere from 6 to 8.8%^5^. Interestingly, the negative effects of radiation on skin have been recognized as a limiting factor in therapeutic radiation exposure levels since the implementation of radiation for clinical benefit in the early 1900s^7^. This direct cutaneous response to radiation has since been defined as acute radiation dermatitis (ARD), and it can have a similar presentation to RRD, though the former results from direct ionizing nuclear damage and perhaps even acute immune cell activation. In fact, up to 95% of patients can experience skin problems due to radiation treatment^8^. While, ARD has been defined by some as a reaction occurring within 30–90 days of radiation exposure, it is likely that many case reports for RRD may be confounded by potential overlaps between ARD^7–10^.

RRD’s specific causes and physiological pathway, however, remain largely unknown. Thus, clinical familiarity and comprehensive repositories of known RRD triggers are of paramount importance.

RRD should be considered in patients presenting with skin changes localized to an area of previous radiation therapy; a biopsy is not needed to confirm the diagnosis and is rarely performed. Triggering drugs may be withdrawn or discontinued, depending on patient preference and severity, to allow for complete resolution of symptoms^11^. In this case, though grade 1-2 dermatologic (radiation recall) and neurologic (mood swings and depression) CTCAE may be managed to allow for continuation of treatment, the patient requested to discontinue medication and has refused further hormonal therapy at present. She remains disease free as of July 2020 based on 3D mammography and continues to follow up with Oncology.

Interestingly, while a dose dependent relationship seems to exist between radiation exposure and potential for RRD, even in separate anatomical sites that receive concurrent and equivalent radiation exposure doses, RRD may occur asymmetrically and not in all sites^2,12,13^.

A thorough investigation of our patient’s history of oncological interventions was crucial for the correlation of the patient’s symptoms and clinical signs to recent initiation of letrozole. This case is the second to identify letrozole as the probable trigger of RRD, and it also highlights the significant time gap that can exist between radiation therapy and the development of RRD. Intervals as long as 15 years between completion of radiation and RRD have been suggested since the 1970s, yet there are not sufficient data regarding the mean time of onset^14^.

Outside of RRD, the differential diagnoses can include more common entities, such as erysipelas, herpes zoster, fungal infection, erysipelatous carcinoma, angiosarcoma, fixed drug reaction, panniculitis, and other radiation reactions^2^. RRD should be favored in patients with a history of radiation, a recurrent or chronic course despite antibiotic intervention, a lack of laboratory evidence of infection, and recent exposure to known trigger drugs, which our case adds to. Since RRD can present with erythema, warmth, and pain, it is commonly misdiagnosed as cellulitis^4,15,16^.

Theories pertaining to the pathogenesis of RRD focus on radiation effects on the skin and characteristics of the trigger drug, since many are anticancer agents; however, this may simply reflect a sampling bias given radiation’s critical utility in treating cancer and the fact that even antibiotics can induce RRD^3^. Some proposed explanations include: depletion of epithelial stem cells and associated cell proliferation impairment, genetic predispositions, increased vascular permeability impacted drug pharmacokinetics, the induced expression of cytokines, and that RRD is a drug hypersensitivity reaction^2,17,18^. Some of these suggestions gain credence as epithelial tissues, including the lungs, esophagus, and gut, are the typical organs that experience RRD^19^. Yet, RRD likely results from a confluence of the above factors or undiscovered ones given trigger drug rechallenge may fail to elicit as pronounced and sometimes no RRD, perhaps due to resident tissue memory^18^.

Our patient’s history definitively correlates letrozole with RRD and extends the known radiation-RRD gap for this drug to up-to nearly 10 years. Significantly, only four other cases of aromatase-inhibitor-induced RRD have been reported, including one for letrozole in a patient with a recent (<3 month) history of radiation (Table 1)^6,20–22^. Of note, in one of these cases, letrozole did not induce RRD while another aromatase inhibitor did. Our case also stresses how even non-cytoxic medications can induce RRD and the need for further research^17^. We believe the architectural changes resultant from our patient’s left mastectomy reiterated epithelial tissue’s unique sensitivity to RRD, as well.

**Table 1:** Cases of aromatase inhibitor induced radiation recall dermatitis (RRD).

Author (Year)	Age/Gender	Radiotherapy Dose (Gy)	Drug/Dose	Time		Treatment	RRD on Rechallenge
				Radiation to RRD	Drug to RRD		
Haydaroglu et al. (2012)	68/F	50 + 10 Gy	Anastrazole/1 mg	2 mo	2 mo	Methylprednisolone aseponate, 10% urea, and fusidic acid creams started; anastrozole discontinued	NA
Foster et al. (2014)	74/F	50.4 + 10 Gy	Letrozole/2.5 mg	2 mo	30 days	Letrozole withdrawn	No
^a^Ioannidis et al. (2014)	58/F	Data not available	Everolimus + Exemestane/10 mg + 25 mg	10 yrs	3 days	Exemestane + everolimus withdrawn; systemic corticosteroids, topical dexpanthenol started	No
^a^Marchand et al. (2016)	74/F	66 Gy	Exemestane/25 mg	<1 yr	27 days	Exemestane discontinued	NA
Present case	78/F	42.56 + 8.1 Gy	Letrozole/2.5 mg	9 yrs	14 days	Letrozole discontinued	NA

^a^These 2 cases were published as Letters to the Editor.

Fortunately, RRD tends to decrease in intensity with each administration of the target drug and resolve with discontinuation of the medication. In some cases of cancer with high rates of recurrence, triggering medication has been continued and RRD has been managed with topical treatments, alone^23^. In most cases, the drug can be continued with symptomatic treatment including systemic or topical steroids and antihistamines^3^. For our patient, the adverse drug side effects in addition to the RRD prompted the discontinuation of letrozole, which resulted in gradual fading of skin changes over the period of 3 months. In summary, the authors hope that this case raises awareness of RRD in oncology patients.

## Methods

### Ethics statement

The patient provided written informed consent to participate in this case report and to the use of their data, including photographs, for publication. IRB approval was not required per Johns Hopkins Medicine institutional guidelines, and all additional relevant ethical considerations were complied with.

### Data collection

Images were collected at clinical visits at Johns Hopkins Hospital or provided to the care team by the patient.

### Reporting summary

Further information on research design is available in the Nature Research Reporting Summary linked to this article.

## Supplementary information

Reporting Summary

## Data Availability

All the data supporting the findings in this case report are contained within the text.
